# Middle cerebral artery pressure laterality in patients with symptomatic ICA stenosis

**DOI:** 10.1371/journal.pone.0245337

**Published:** 2021-01-08

**Authors:** Madelene Holmgren, Karen-Helene Støverud, Laleh Zarrinkoob, Anders Wåhlin, Jan Malm, Anders Eklund

**Affiliations:** 1 Department of Radiation Sciences, Umeå University, Umeå, Sweden; 2 Department of Clinical Science, Neurosciences, Umeå University, Umeå, Sweden; 3 Department of Surgical and Perioperative Sciences, Umeå University, Umeå, Sweden; 4 Umeå Center for Functional Brain Imaging, Umeå University, Umeå, Sweden; Universidade de Lisboa Instituto Superior Tecnico, PORTUGAL

## Abstract

An internal carotid artery (ICA) stenosis can potentially decrease the perfusion pressure to the brain. In this study, computational fluid dynamics (CFD) was used to study if there was a hemispheric pressure laterality between the contra- and ipsilateral middle cerebral artery (MCA) in patients with a symptomatic ICA stenosis. We further investigated if this MCA pressure laterality (ΔP_MCA_) was related to the hemispheric flow laterality (ΔQ) in the anterior circulation, i.e., ICA, proximal MCA and the proximal anterior cerebral artery (ACA). Twenty-eight patients (73±6 years, range 59–80 years, 21 men) with symptomatic ICA stenosis were included. Flow rates were measured using 4D flow MRI data (PC-VIPR) and vessel geometries were obtained from computed tomography angiography. The ΔP_MCA_ was calculated from CFD, where patient-specific flow rates were applied at all input- and output boundaries. The ΔP_MCA_ between the contra- and ipsilateral side was 6.4±8.3 mmHg (p<0.001) (median 3.9 mmHg, range -1.3 to 31.9 mmHg). There was a linear correlation between the ΔP_MCA_ and ΔQ_ICA_ (r = 0.85, p<0.001) and ΔQ_ACA_ (r = 0.71, p<0.001), respectively. The correlation to ΔQ_MCA_ was weaker (r = 0.47, p = 0.011). In conclusion, the MCA pressure laterality obtained with CFD, is a promising physiological biomarker that can grade the hemodynamic disturbance in patients with a symptomatic ICA stenosis.

## Introduction

**Fig 1:**
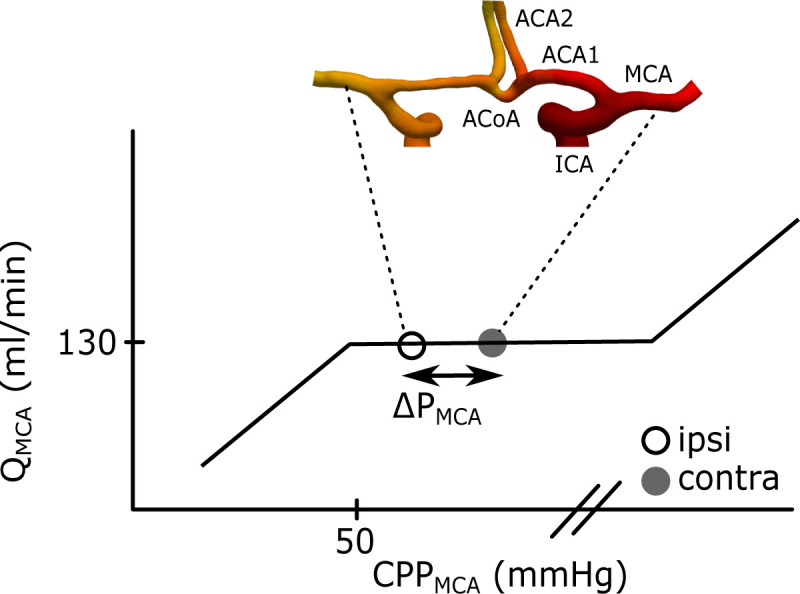
Illustration of the ΔP_MCA_ between the contra- and ipsilateral MCA. The curve corresponds to Lassen’s autoregulation curve and is a schematic relationship between the Q_MCA_ and the cerebral perfusion pressure (CPP_MCA_) in MCA. The CPP_MCA_ is here defined as the mean arterial pressure in MCA minus the intracranial pressure. This means that MCA should have a maintained flow within a range of perfusion pressures at the MCAs. The separation between the contra- and ipsilateral CPP_MCA_ was defined as ΔP_MCA_. The geometry is an example of the anterior part of CW. Left and right ICA branches into the left and right MCA and ACA1. The two ACA1s is connected by the ACoA before they branch into the left and right ACA2. The posterior circulation is not displayed in the figure, but the anterior and the posterior part is connected via the posterior communicating arteries.

An internal carotid artery (ICA) stenosis is a major risk factor for a transient ischemic attack (TIA) or a stroke. In addition to the embolization risk [1, 2], a severe stenosis (70–99%) may cause abnormal pressure and flow asymmetries in the cerebral arterial tree [3]. As a consequence, collaterals in the circle of Willis (CW) are recruited to maintain sufficient flow to the side with the symptomatic stenosis (ipsilateral) [4, 5]. The cerebral autoregulation of the cerebrovascular resistance further works to maintain a constant total cerebral blood flow for varying cerebral perfusion pressures, as described by Lassen’s autoregulatory curve [6]. Correspondingly, we assumed that this also holds locally for each vascular territory, such as the middle cerebral artery (MCA) territory (Fig 1). A hemispheric MCA pressure laterality may therefore be viewed as a measure of the separation between the two MCAs on the Lassen curve (Fig 1). This implies that the first sign of hemodynamic disturbance should be observed as a laterality in pressure, and not in flow. We defined this separation as a hemodynamic MCA pressure disturbance. Thus, there is potentially a gain in analyzing degree of stenosis, flow disturbance and pressure laterality independently to fully understand the hemodynamic disturbance from a carotid stenosis.

Measurements of the pressure in cerebral arteries would require highly invasive techniques. With computational fluid dynamics (CFD), relative pressures can instead be modelled non-invasively [7, 8]. Based on medical images, patient-specific arterial geometries and flow boundary conditions are obtained [9–11]. Previously, CFD analysis of the cerebral arterial system has mainly used flow or pressure boundary conditions at inlets and assumption about pressures at outlets [7, 9, 10, 12]. By using 4D flow MRI, which simultaneously assesses flow rates in all cerebral arteries [13–15], we evaluate the feasibility of a set-up with flow conditions at all inlet and outlet boundaries [16]. Thereby, we avoid assumptions about vascular resistances and outlet pressures. This gives patient-specific reliable flow distributions and a possibility to estimate the hemispheric pressure laterality between MCAs.

The aim of this study was to use patient-specific flow rates from 4D flow MRI and geometries from computed tomography angiography (CTA) together with CFD analysis, to detect the MCA pressure laterality. Second, we aimed to investigate its relation to the hemispheric flow imbalance, and its potential as an early marker of the hemodynamic pressure disturbance in patients with symptomatic ICA stenosis.

## Materials and methods

### Model assumption about circle of Willis

**Fig 2:**
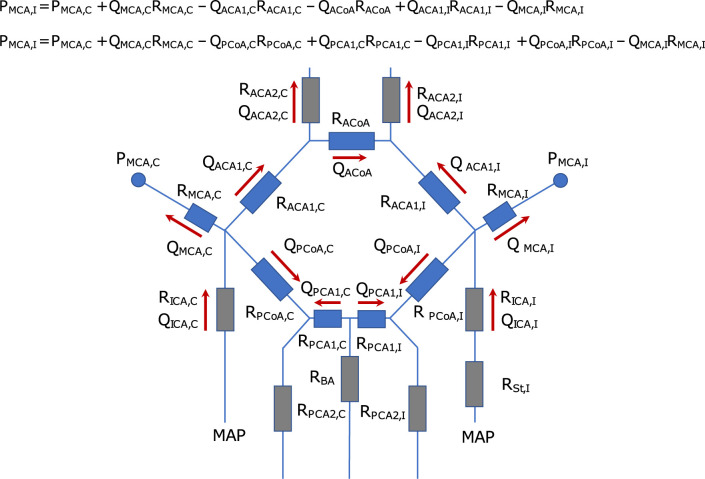
Circuit analogy of the circle of Willis. The figure illustrates a schematic of the CW, denoting the ipsi (I)- and contralateral (C) side of the ICA stenosis (St). It is modelled as an electrical circuit, where the change in pressure across each artery is related to the flow (Q) and the vascular resistance (R) of the artery. The arrows indicate the normal flow direction in each artery, and the circles indicate the two pressure sites for the ipsi- and contralateral MCA pressure (P_MCA_). The two equations for P_MCA,I_ describe how the pressure changes, starting at P_MCA,C_, across the anterior collateral pathway versus the posterior collateral pathway. They show that the pressure laterality between the ipsi- and contralateral MCA must be the same for both pathways. Note that the MCA resistance only refers to the arterial segment from the bifurcation to the location of the pressure measurement.

For the CFD simulations, we assumed it sufficient to segment and analyze the anterior part of the CW in order to obtain reliable estimates on the hemispheric MCA pressure laterality. To motivate and exemplify this, we used a circuit analogy (Fig 2). The CW can be represented as a lumped model of an electrical circuit, where the change in pressure across each artery is related to the flow (Q) and the vascular resistance (R) of the artery (Fig 2). If we want to determine the pressure laterality between the ipsi- and contralateral MCA, we can follow the circuit across the anterior pathway, via the proximal anterior cerebral arteries (ACA1) and the anterior communicating artery (ACoA) (i.e., MCA→ACA1→ACoA→ACA1→MCA). Alternatively, across the posterior pathway, via the posterior communicating arteries (PCoA) and the proximal posterior cerebral artery (PCA1) (i.e., MCA→PCoA→PCA1→PCA1→PCoA→MCA). The pressure change across each individual artery is different, but the total sum of pressure changes between the ipsi- and contralateral MCA must be the same for both pathways. Thus, although both the anterior and the posterior circulation contributes to the overall resistance between the left and right hemisphere, analysis of only one pathway is required to determine the pressure laterality. Correspondingly, for estimating the MCA pressure laterality with CFD, the use of only the anterior or posterior pathway should be sufficient. The anterior pathway offers larger vessels and higher flow rates compared to the posterior pathway, which makes the anterior pathway the most feasible choice. In summary, CFD simulations using the anterior pathway for estimating an MCA pressure laterality should be valid as long as the correct flow rates and geometries of the MCA, ACA1 and ACoA arteries are used.

The correct flow in MCA, ACA1 and the ACoA was accomplished by the boundary conditions of the CFD model (See section about boundary conditions). These conditions required segmentation of the left and right ICA, MCA, ACA1, ACoA and the distal anterior cerebral artery (ACA2). Based on the model assumption, the posterior circulation and the ophthalmic arteries were removed.

### Patients

This study was based on a patient cohort previously described, where 38 TIA or ischemic stroke patients with a symptomatic ICA stenosis were included and investigated with 4D flow MRI [3]. For the current study, 10 of these patients were excluded because of no CTA investigation (n = 7), insufficient image quality (n = 2) and a missing ACA1 (n = 1). The final study population thus consisted of 28 patients (73±6 years, range 59–80 years, 21 men) with a symptomatic ICA stenosis ≥50% or occlusion (n = 1) with or without a non-symptomatic contralateral stenosis. The mean degree of stenosis on the ipsi- and contralateral side of the symptomatic stenosis was 76±12% and 38±26%, respectively, graded according to the North American Symptomatic Carotid Endarterectomy Trial (NASCET) [17]. The mean modified Ranking Scale (mRS) score was 0 (range 0–1) and the mean NIHSS score was 1 (range 0–6). The systolic and diastolic blood pressure was 138±17 mmHg and 70±10 mmHg, respectively, with a mean arterial pressure (MAP) of 93±11 mmHg. The heart rate during the MRI was 64±11 bpm. Risk factors were diabetes mellitus (25%), hyperlipidemia (54%), hypertension (75%) and ever smoker (64%).

The ethical review board of Umeå University (Dnr: 2011-440-31M) and the Swedish Ethical Review Authority (Dnr: 2019–05909) approved the study. It was performed in accordance with the guidelines of the Declaration of Helsinki. Oral and written information about the study was given to all participants and written consent was obtained from all participants.

### Imaging

For flow rate measurements, MRI scans were performed on a 3T scanner (GE Discovery MR 750, Milwaukee, WI, USA) with a 32-channel head coil. With a scan time of about nine minutes, the 4D flow MRI data was acquired with a balanced 5-point phase contrast vastly undersampled isotropic projection reconstruction (PC-VIPR) sequence with full brain coverage [15, 18]. Scan parameters were: TR/TE 6.5/2.7 ms, Venc 110 cm/s, flip angle 8°, 16000 radial projections, acquisition resolution 300×300×300, imaging volume 220×220×220 mm^3^, reconstructed resolution 320×320×320, isotropic voxel size 0.7×0.7×0.7 mm^3^, 20 reconstructed cardiac time-frames. An angiographic complex difference image and velocity images in three directions were reconstructed.

Flow rate quantification was performed according to a previously developed post-processing method [19]. Pulsatile blood flow rates were measured in the left and right ICA, proximal middle cerebral artery (MCA1), ACA1 and the ACA2. There was no measurable flow in three ACA1 and four ICA on the ipsilateral side. Flow rates were obtained by averaging the flow waveforms from 15 consecutive cut planes [19].

Patient-specific vessel geometries were obtained from the clinical CTA images, from different hospitals. The trans-axial image resolution ranged between 0.33 to 0.625 mm, and the reconstructed slice thickness ranged between 0.3 to 0.625 mm.

### Geometries and mesh generation

Before segmentation, CTA images were cropped to only include the CW. In axial direction, ICAs were cut at the C3-C4 segment and the distal anterior cerebral arteries at ACA3 level. In left/right direction, the MCAs were cut to include MCA2. We included MCA2, ACA3 and the relatively long ICAs instead of adding flow extensions to the geometry. The segmentations were performed with Synopsys’ Simpleware™ software (ScanIP P-2019.09; Synopsys, Inc., Mountain View, USA).

Images were resampled with linear interpolation to obtain an isotropic voxel size of 0.3 or 0.3125 mm, depending on original resolution. Background noise from surrounding tissue was reduced by a bilateral filter. For vessel wall detection, a mask was generated with a threshold filter, followed by the software specific gradient-based filter ‘Local surface correction’. Before model generation, a volume and topology preserving smoothing filter was applied.

**Fig 3:**
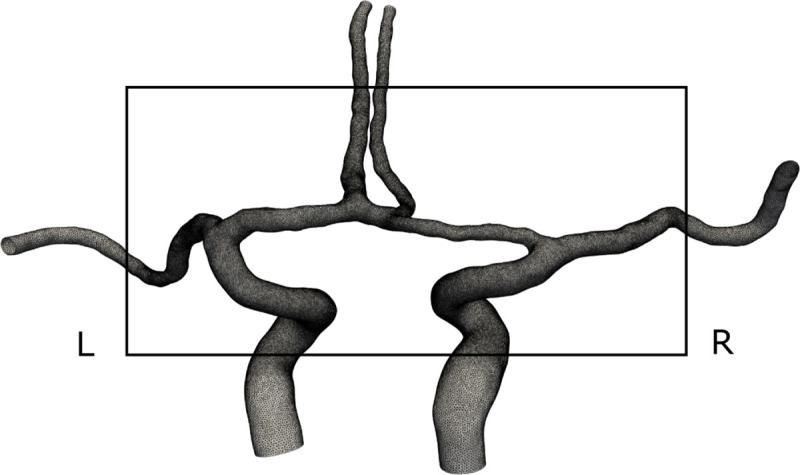
A computational geometry and mesh from a representative patient. From the posterior view, with left and right denoted in the figure. Note how only the anterior part of the CW was included in the model, according to our model assumption. The black square shows the mesh refinement region of our simulations. Longer segments of the arteries were included as flow extensions.

All meshes were generated with the Simpleware FE module and imported as meshes into COMSOL Multiphysics® (COMSOL Multiphysics®, version 5.4, www.comsol.com, COMSOL AB, Stockholm, Sweden). In the refinement region of our simulations, i.e., across the MCA1/ACA1 bifurcations (Fig 3), our target edge length was equal to the interpolated voxel size. Outside the refined region, the target edge length was increased to 1.5 times the voxel size. The meshes included boundary layers with four sublayers and a total target width of approximately 0.14 mm [20]. The total number of elements ranged between 0.9–1.6 million, and the average edge length of all elements was 0.17 mm.

A coarser and a finer mesh, without any refinement regions, was used to investigate the mesh sensitivity of the stationary solutions. For the coarse mesh, the target edge length was twice the voxel size and for the fine mesh it was equal to the voxel size. The target boundary layer thickness was 0.2 and 0.1 mm, respectively. The total number of volume elements ranged between 0.5–1.1 and 2.7–5.5 million elements for the coarser and the finer mesh, respectively.

### Computational fluid dynamics

Navier-Stokes equations were solved with the CFD module in COMSOL Multiphysics®. Blood was assumed to be an incompressible Newtonian fluid with density *ρ* = 1060 kg/m^3^ and viscosity *μ* = 3.45 mPa·s. The flow was assumed laminar and the vessel walls impermeable and rigid. Primary simulations with stationary flows were performed for all of the 28 patients.

Time-resolved simulations with pulsatile flow waveforms were performed in 24 of 28 patients. One patient was excluded because of convergence problems and three because of MR reconstruction problems. Time-dependent simulations were run for two cardiac cycles, with a linear interpolation between each of the 20 cardiac phases, and 4000 time steps per second. The initial flow condition was ramped up from 0 to the flow rate in the first cardiac phase during about 0.5 s, followed by the full cardiac phases. The second cardiac cycle was used in the analysis. A pilot simulation with four cycles indicated two cycles to be sufficient.

### Boundary conditions

There were totally six inlet and outlet boundaries in the CFD model. Inlet boundaries were the left and right ICA. Outlet boundaries were the left and right MCA and ACA2. At each inlet or outlet boundary, we applied a laminar parabolic flow rate profile, using COMSOL’s build-in boundary condition for fully developed flow. A no-slip condition was applied to the vessel walls. For mass conservation within the model, and to achieve the measured flow in MCA and ACA1, the flow rate boundary conditions were defined as follows:

- The inlet boundary in each ICA was calculated from the summed measured flow rates of MCA1 and ACA1 on the same side.
- The outlet boundary in each MCA was set as the measured flow rate.
- The right ACA2 outlet was calculated by (ACA2_R_ / ACA2_tot_)*ACA1_tot_.
- The left ACA2 outlet was calculated by (ACA2_L_ / ACA2_tot_)*ACA1_tot_.

The ACA1_tot_ and ACA2_tot_ was the sum of the measured flow in the left and right ACA1 and ACA2, respectively. Thus, the proportional difference between the measured flow rates in ACA2 was used as boundary condition.

Since the pressure is only uniquely defined up to a constant when flow rates are applied at all boundaries, i.e., Dirichlet condition, the pressure level was set as a zero-reference pressure point condition at the contralateral ICA inlet boundary.

### Pressure and flow laterality

The hemispheric MCA pressure laterality (ΔP_MCA_) was defined as the pressure difference between the contra- and ipsilateral MCA1, where ipsilateral is the side of the symptomatic stenosis. The goal was to measure the average pressure at a distance of 5 mm from the center of the MCA1/ACA1 bifurcation. For the time-resolved simulations, the pulsatile pressure waveforms of the contra- and ipsilateral MCA1 were averaged and subtracted to give the ΔP_MCA_.

The hemispheric flow rate laterality (ΔQ) was defined as the flow rate difference between the contra-and ipsilateral ICA, MCA1 and ACA1, respectively, measured in the 4D flow MRI data.

Based on findings in a previous modelling study and measurements in canines, it has been shown that a degree of stenosis >50% can generate ICA pressure drops higher than 5 mmHg [21, 22]. This motivated us to set a 5 mmHg MCA pressure laterality as the lower limit of a potentially clinical interesting difference.

### Statistical analysis

All mean values were presented as the mean±standard deviation. A one-sample Kolmogorov-Smirnov test was used for normality testing. Wilcoxon signed rank test was used to test for all significant differences. Pearson’s linear correlation coefficient was used for all reported correlations. The significance level was set as p<0.05. All statistical analysis and MRI flow rate measurements were performed with MATLAB (R2019a, MathWorks, Natick, MA).

## Results

### MCA pressure laterality

**Fig 4:**
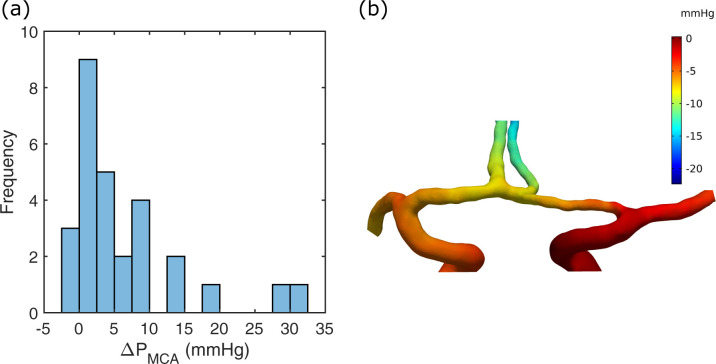
MCA pressure laterality. (a) Histogram of ΔP_MCA_ for the patient group. (b) The CFD pressure of a stationary simulation for the case in Fig 3. A zero-reference pressure was set at the contralateral ICA, which was the right ICA inlet in this case. The ΔP_MCA_ was 3.0 mmHg in this example.

In the 28 patients, the hemispheric MCA pressure laterality (ΔP_MCA_) between the contra-and ipsilateral side was 6.4±8.3 mmHg (p<0.001) (median 3.9 mmHg, range -1.3 to 31.9 mmHg) (Fig 4A). Degree of ipsilateral ICA stenosis was not correlated to ΔP_MCA_ (r = 0.23, p = 0.23). When dividing patients into those with or without a contralateral ICA stenosis ≥50%, the ΔP_MCA_ was 5.5±5.1 mmHg and 6.9±9.6 mmHg, respectively. In Fig 4B, the pressure from the patient in Fig 3 is shown. The pressure result for each patient is found in S1 Fig.

The mean difference in ΔP_MCA_ between the stationary and the time-resolved simulations was -0.14±0.26 mmHg (p = 0.029), and the correlation was r = 0.99 (p<0.001). The systolic and diastolic ΔP_MCA_ was 9.9±12.8 (p<0.001) and 3.4±6.2 (p = 0.029), respectively. In the subsequent analysis, only pressures from the stationary simulations are used.

### Collateral flow relationship to pressure

**Fig 5:**
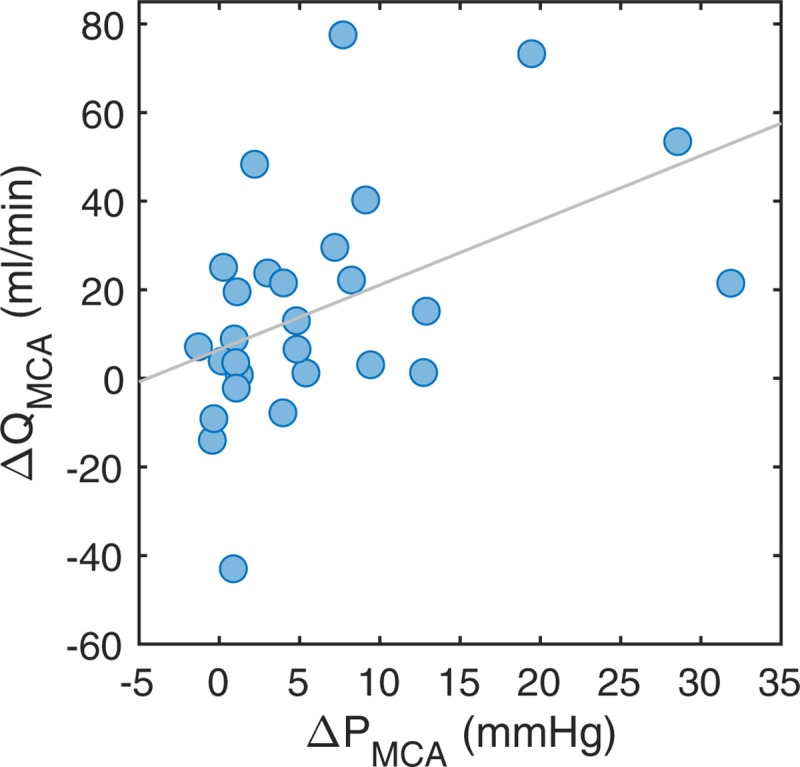
MCA flow relationship to pressure. The ΔQ_MCA_ versus the ΔP_MCA_. The linear correlation was r = 0.47 (p = 0.011).

There was a correlation between ΔP_MCA_ and the MCA flow laterality (ΔQ_MCA_) (r = 0.47, p = 0.011) (Fig 5). When ΔP_MCA_ was <5mmHg, there was no MCA flow laterality (6.2±19.8 ml/min, p = 0.14, n = 17). With ΔP_MCA_>5mmHg, there was an MCA flow reduction on the ipsilateral side (30.8±27.4 ml/min, p<0.001, n = 11).

**Fig 6:**
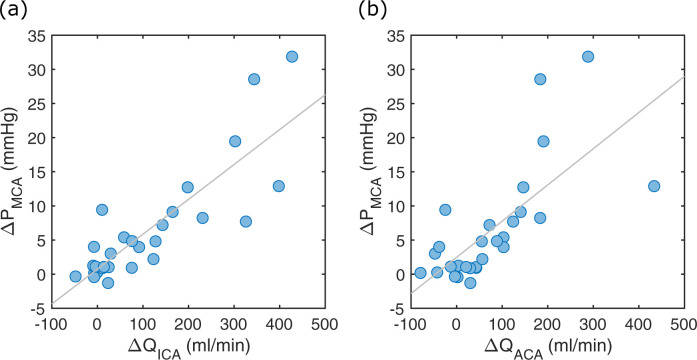
ICA and ACA flow relationship to pressure. (a) The ΔP_MCA_ versus ΔQ_ICA_. The linear correlation was r = 0.85 (p<0.001). (b) The ΔP_MCA_ versus ΔQ_ACA_. The linear correlation was r = 0.71 (p<0.001).

There was a significant hemispheric flow rate laterality between the contra- and ipsilateral ICA, MCA1 and ACA1, respectively (Table 1). The flow rate laterality between ICA (ΔQ_ICA_) and ACA1 (ΔQ_ACA_) was highly correlated (r = 0.90, p<0.001). The ΔQ_ICA_ was correlated to ΔP_MCA_ (r = 0.85, p<0.001) (Fig 6A). Similarly, there was a strong relationship between the ΔQ_ACA_ and ΔP_MCA_ (r = 0.71, p<0.001) (Fig 6B).

**Table 1:** Geometry and flow rate features (mean±standard deviation).

Artery	D (mm)	Q (ml/min)	ΔQ (ml/min)	Re	Wo
ICA^a^	3.8±0.74	193±98	111±137 (p<0.001)	363±169	2.7±0.51
MCA1^b^	2.6±0.36	130±27	16±26 (p = 0.002)	332±69	1.8±0.26
ACA1^a^	1.9±0.30	72±68	75±111 (p = 0.001)	291±153	1.3±0.20

D: diameter; Q: flow rate; ΔQ: hemispheric flow rate laterality; Re: Reynolds number; Wo: Womersley number; ICA: internal carotid artery; MCA1: proximal middle cerebral artery; ACA1: proximal anterior cerebral artery. All flow rates were measured from 4D flow MRI data and all diameters from CTA. The significance level was set at p<0.05.

^a^ Estimated from the whole branches.

^b^ Estimated from 5 mm long segments.

### Mesh sensitivity

The mesh sensitivity analysis revealed a high concordance. The mean difference in ΔP_MCA_ between the coarser and the intermediate mesh was 0.06±0.36 mmHg (p = 0.68) with a correlation of r = 0.99 (p<0.001). For the finer mesh, the mean difference in ΔP_MCA_ against the intermediate mesh was 0.16±0.35 mmHg (p = 0.027) with a correlation of r = 0.99 (p<0.001).

## Discussion

We assessed hemispheric MCA pressure laterality (ΔP_MCA_) as a potential marker of early hemodynamic perfusion pressure disturbance. The ΔP_MCA_ was estimated in patients with a symptomatic ICA stenosis by CFD analysis, using CTA vessel geometries of the anterior CW, together with blood flow rates from 4D flow MRI. The analysis revealed a large variation in ΔP_MCA_. Although many patients had a balanced pressure laterality, about 40% had a hemodynamic pressure disturbance, if we define this as a laterality of >5 mmHg. In the future, clinical applications of the ΔP_MCA_ can be patient selection and preoperative planning of carotid surgery or stenting, or to identify hemodynamic disturbances in patients with an asymptomatic stenosis or patients with ICA stenosis at high risk for hypoperfusion.

### Collateral recruitment and perfusion pressure

Without an ICA stenosis, we expect a pressure balance between the left and right MCA. Therefore, ΔP_MCA_ can be interpreted as the degree of primary collateral system recruitment. Furthermore, this holds with or without an affected ΔQ_MCA_, since the ipsilateral flow can be maintained by the autoregulated downstream vasodilatation [23–25]. The ΔP_MCA_ is visualized as an increased separation between pressures in the MCA Lassen curve (Fig 1). A further increase of the stenosis severity, with an exceeded vasodilatory compensation, could develop a critical blood flow reduction and eventually MCA territory hypoperfusion as well as reduced capacity for washout of emboli [26, 27]. The weak relationship between ΔQ_MCA_ and ΔP_MCA_ indicates that this patient group, in general, was on the plateau part of the MCA Lassen curve. However, for the group with ΔP_MCA_>5 mmHg there was a significant ΔQ_MCA_, indicating that the ipsilateral MCA pressures have decreased towards the flow-reduction region of the curve. For the ΔP_MCA_<5 mmHg group, the compensatory capability to maintain MCA flow through collateral recruitment and autoregulatory response was essentially sufficient. A natural MCA flow laterality, due to hemispheric differences in size of vascular territories, challenge the use of MCA flow laterality as an indication of disturbed hemodynamics. A corresponding natural variability in pressure laterality is not expected. Avoiding natural variability and having an interval of a stenosis induced pressure laterality with autoregulatory compensated MCA blood flow, supports that ΔP_MCA_ has a potential as an early marker for detecting the hemodynamic pressure disturbance.

The ΔP_MCA_ was highly correlated to the ΔQ_ICA_, demonstrating that the stenosis causes the pressure imbalance. This pressure imbalance was expected since a stenosis is causing a recruitment of the collateral system [3, 25]. If ΔP_MCA_ is an important indicator of disturbed hemodynamics, as suggested in our study, and the CFD analysis to estimate it is unavailable, then the ICA flow laterality is the most reliable indication of this with respect to flow. This is of clinical importance since the degree of stenosis is alone insufficient to characterize the hemodynamic disturbance [23, 28].

### Hemodynamic analysis with CFD

A main goal with our CFD model was to incorporate the patient-specific 4D flow MRI measurements and to guarantee a correct flow distribution across the anterior part of the CW. Although patient-specific boundary conditions are important [29, 30], flow outlet boundaries are rare in CFD analysis of CW [8, 16, 31]. A challenge for resistive or pressure outlet boundary condition strategies is to correctly represent the highly resistant capillary bed, which dominates the resistance in the larger arteries. Lumped parameter models or pressures are commonly used instead, but these still require assumptions about territorial resistances [7, 10, 12]. By the model design with flow boundary conditions, we avoided assumptions about territorial vascular resistance and outlet pressures.

Since the posterior circulation of the CW was excluded, the model was limited to patients with an anterior circulation with all arteries visible. Our method should give the correct ΔP_MCA_ as long as flows and vessel geometries for MCA, ACA1 and ACoA are correct. For patients with bilateral stenoses, the MCA pressure was probably decreased on both sides, potentially reducing the ΔP_MCA_, in spite of a distinct bilateral hemodynamic disturbance [32]. For these patients, collaterals might have been recruited from the posterior circulation [28, 33], and a similar CFD analysis between MCA and PCA perfusion pressure could be useful.

The comparison of stationary and time-resolved simulations showed that it was sufficient to use the stationary case to study the mean pressure distribution in the CW. Time-resolved simulations, however, offers the possibility to investigate the pulsatile component of arterial blood flow. Womersley numbers of the major arteries were borderline with respect to using a Womersley profile in the pulsatile analysis [34]. However, we assumed that the effect of these profiles was small and that the inlets, functioning as flow extensions, reduced the boundary effects. A limitation for the time-resolved simulations was the assumption about rigid walls, which excluded dampening effects from the arterial walls [19]. The main advantage of the stationary simulation was that it was performed on a standard PC in less than an hour, while the time-resolved results required up to four days of computational time.

The mesh sensitivity study showed a statistically significant difference in ΔP_MCA_ of about 2.5% between the intermediate and the finer mesh, but the difference was small and not considered physiologically important in our analysis. Pressure drop is highly correlated to arterial diameters and our results are therefore sensitive to the segmentation. The ACA1 diameters in our study were in agreement with previous studies [32, 35, 36].

### Clinical applications

The lower pressure limit for the cerebral autoregulatory function has been shown to have a considerable inter-subject variability [37]. The variability contradicts the use of group defined thresholds in clinical guidance for avoiding hypoperfusion, and rather motivates the use of intra-subject approaches. This is what we have done, when we assumed a normotensive contralateral side and identifies the deviation on the ipsilateral side.

A determined ΔP_MCA_ can give an insight into the consequences of having a large ICA stenosis or occlusion, where a low cerebral perfusion or hypoperfusion may increase the risk for ischemic stroke [27, 38]. The findings suggest that ΔP_MCA_ could be an early sign of hemodynamic pressure disturbance in patients with ICA stenosis, observed before MCA flow declines and hypoperfusion symptoms occurs. Specifically, this could be of clinical importance for asymptomatic patients [39], where indications for surgery is not well established, but this has to be confirmed in larger prospective studies.

Another situation for determining perfusion pressure to the brain, is when considering risk for intraoperative hypoperfusion during carotid intervention [40, 41]. Estimation of ΔP_MCA_ could be important in the planning of surgery, since a reduced perfusion pressure is a risk when the ICA is clamped, to temporarily interrupt the ICA blood flow during the intervention, and ΔP_MCA_ gives a strong indication of the current hemodynamic pressure disturbance and the collateral capacity.

### Limitations

The main limitation was the lack of control group. However, this was a relatively large patient-group for a CFD study with stroke patients and the range of pressure lateralities showed a distribution within the group of patients with ICA stenosis. A control group would have added knowledge about the expected variation without a stenosis. Additionally, the stroke patients recruited for the study were limited to those eligible for ICA surgery or MRI, which excluded asymptomatic cases and patients with the most severe stroke symptoms. Ischemic regions could potentially increase the MCA flow laterality, while the effect on pressure laterality should decrease. From the data in Table 1, we found that the MCA flow laterality was about 12% of the mean MCA flow. For the corresponding average perfusion pressure, we used the approximate MAP of 93 mmHg and assumed an intracranial pressure of 11.6 mmHg [19]. The mean pressure laterality found in our paper was about 8% of this approximated cerebral perfusion pressure. The relative pressure laterality is therefore lower compared to the relative flow laterality. In addition to the effect of autoregulation, the reason for the inconsistency could be that lesioned tissue reduces the needed blood flow and increased the resistance on the symptomatic side. The volumes of the ischemic regions were not measured in the included patients, but the low mRS and NIHSS scales indicated that these volumes should have been small.

The accuracy of the CFD results was primarily limited by the imaging modality [29, 42]. We used two different image modalities, with scans separated in time, which entails a potential risk for geometry discrepancy. An option was to only use the MRI based data. This is, however, sensitive to low or absent flow and would have caused potential vessel-area inaccuracies [43–45]. Therefore, the CTA data was considered a better alternative for our analysis.

The accuracy of the pressure distributions is also limited by the accuracy of the 4D flow MRI measurements and the segmentation method. We can expect some deviations, caused by partial volume effects, small branches along the arteries and random errors between repeated measurements. We tried to reduce these errors by using a flow segmentation method which we have shown is less sensitive to different cross-sectional areas and flow rates [13]. In addition, 4D flow MRI is likely less affected by partial volume effects compared to 2D PC-MRI because of its reduced inflow effects causing more tissue-like magnitude in the blood. The variability along the arteries was managed by averaging multiple of subsequent cut planes, as a representation of the average flow in each artery. We have found this to be important for noise reduction [19]. However, the random variation within repeated measurements is always a remaining source of variability, caused by the true physiological variations of flow in cerebral arteries [13].

## Conclusions

This study presents a feasible method for CFD analysis with patient-specific flow rates from 4D flow MRI and CTA based geometries. In patients with symptomatic ICA stenosis, there was a significant ΔP_MCA_, but the disturbance was not strongly related to ΔQ_MCA_. This shows that measurements of MCA blood flow were not sufficient to detect lateral intracranial perfusion pressure drops from an ICA stenosis. MCA pressure laterality can be a promising physiological biomarker of hemodynamic disturbance in patients with ICA stenosis, motivating investigation of its clinical importance in a prospective clinical trial.

## Supporting information

S1 Dataset Pressure lateralities and flow rates used to build graphs. (XLSX)

S1 Fig CFD pressure results. The simulated pressures for each geometry of the 28 patients. The scale might differ between images.
(PDF)

## References

[1] Howard DPJ, van Lammeren GW, Rothwell PM, Redgrave JN, Moll FL, de Vries JPPM, et al Symptomatic carotid atherosclerotic disease: Correlations between plaque composition and ipsilateral stroke risk. Stroke. 2015;46:182–9. 10.1161/STROKEAHA.114.007221 25477221 PMC4285579

[2] Grotta JC. Carotid Stenosis. N Engl J Med. 2013;369:1143–50. 10.1056/NEJMcp1214999 24047063

[3] Zarrinkoob L, Wåhlin A, Ambarki K, Birgander R, Eklund A, Malm J. Blood Flow Lateralization and Collateral Compensatory Mechanisms in Patients With Carotid Artery Stenosis. Stroke. 2019;50:1081–8. 10.1161/STROKEAHA.119.024757 30943887 PMC6485302

[4] Hendrikse J, Eikelboom BC, van der Grond J. Magnetic resonance angiography of collateral compensation in asymptomatic and symptomatic internal carotid artery stenosis. J Vasc Surg. 2002;36:799–805. 10.1067/mva.2002.127346 12368718

[5] Liebeskind DS. Collateral circulation. Stroke. 2003;34:2279–84. 10.1161/01.STR.0000086465.41263.06 12881609

[6] Lassen NA. Cerebral blood flow and oxygen consumption in man. Physiol Rev. 1959;39:183–238. 10.1152/physrev.1959.39.2.183 13645234

[7] Liu J, Yan Z, Pu Y, Shiu W-S, Wu J, Chen R, et al Functional assessment of cerebral artery stenosis: A pilot study based on computational fluid dynamics. J Cereb Blood Flow Metab. 2017;37:2567–76. 10.1177/0271678X16671321 27702878 PMC5531352

[8] Long Q, Luppi L, König CS, Rinaldo V, Das SK. Study of the collateral capacity of the circle of Willis of patients with severe carotid artery stenosis by 3D computational modeling. J Biomech. 2008;41:2735–42. 10.1016/j.jbiomech.2008.06.006 18674765

[9] Liu X, Gao Z, Xiong H, Ghista D, Ren L, Zhang H, et al Three-dimensional hemodynamics analysis of the circle of Willis in the patient-specific nonintegral arterial structures. Biomech Model Mechanobiol. 2016;15:1439–56. 10.1007/s10237-016-0773-6 26935302

[10] Grinberg L, Cheever E, Anor T, Madsen JR, Karniadakis GE. Modeling blood flow circulation in intracranial arterial networks: A comparative 3D/1D simulation study. Ann Biomed Eng. 2011;39:297–309. 10.1007/s10439-010-0132-1 20661645

[11] Cebral JR, Putman CM, Alley MT, Hope T, Bammer R, Calamante F. Hemodynamics in normal cerebral arteries: qualitative comparison of 4D phase-contrast magnetic resonance and image-based computational fluid dynamics. J Eng Math. 2009;64:367–78. 10.1007/s10665-009-9266-2 19684874 PMC2726749

[12] Alnæs MS, Isaksen J, Mardal KA, Romner B, Morgan MK, Ingebrigtsen T. Computation of hemodynamics in the circle of Willis. Stroke. 2007;38:2500–5. 10.1161/STROKEAHA.107.482471 17673714

[13] Dunås T, Holmgren M, Wåhlin A, Malm J, Eklund A. Accuracy of blood flow assessment in cerebral arteries with 4D flow MRI: Evaluation with three segmentation methods. J Magn Reson Imaging. 2019;50:511–8. 10.1002/jmri.26641 30637846 PMC6767555

[14] Rivera-Rivera LA, Turski P, Johnson KM, Hoffman C, Berman SE, Kilgas P, et al 4D flow MRI for intracranial hemodynamics assessment in Alzheimer’s disease. J Cereb Blood Flow Metab. 2016;36:1718–30. 10.1177/0271678X15617171 26661239 PMC5076787

[15] Gu T, Korosec FR, Block WF, Fain SB, Turk Q, Lum D, et al PC VIPR: A high-speed 3D phase-contrast method for flow quantification and high-resolution angiography. Am J Neuroradiol. 2005;26:743–9. 15814915 PMC7977085

[16] Berg P, Stucht D, Janiga G, Beuing O, Speck O, Thévenin D. Cerebral blood flow in a healthy circle of Willis and two intracranial aneurysms: Computational fluid dynamics versus four-dimensional phase-contrast magnetic resonance imaging. J Biomech Eng. 2014;136:1–9. 10.1115/1.4026108 24292415

[17] North american symptomatic carotid endarterectomy trial: Methods, patient characteristics, and progress. Stroke. 1991;22:711–20. 10.1161/01.str.22.6.711 2057968

[18] Johnson KM, Markl M. Improved SNR in phase contrast velocimetry with five-point balanced flow encoding. Magn Reson Med. 2010;63:349–55. 10.1002/mrm.22202 20099326 PMC3418793

[19] Holmgren M, Wåhlin A, Dunås T, Malm J, Eklund A. Assessment of cerebral blood flow pulsatility and cerebral arterial compliance with 4D flow MRI. J Magn Reson Imaging. 2020;51:1516–25. 10.1002/jmri.26978 31713964 PMC7216927

[20] Evju Ø, Pozo JM, Frangi AF, Mardal KA. Robustness of common hemodynamic indicators with respect to numerical resolution in 38 middle cerebral artery aneurysms. PLoS One. 2017;12:1–15. 10.1371/journal.pone.0177566 28609457 PMC5469453

[21] Lal BK, Beach KW, Sumner DS. Intracranial collateralization determines hemodynamic forces for carotid plaque disruption. J Vasc Surg. 2011;54:1461–71. 10.1016/j.jvs.2011.05.001 21820834

[22] Turk AS, Johnson KM, Lum D, Niemann D, Aagaard-Kienitz B, Consigny D, et al Physiologic and anatomic assessment of a canine carotid artery stenosis model utilizing phase contrast with vastly undersampled isotropic projection imaging. Am J Neuroradiol. 2007;28:111–5. 17213435 PMC8134124

[23] Shakur SF, Hrbac T, Alaraj A, Du X, Aletich VA, Charbel FT, et al Effects of extracranial carotid stenosis on intracranial blood flow. Stroke. 2014;45:3427–9. 10.1161/STROKEAHA.114.006622 25228258

[24] Derdeyn CP, Grubb RL, Powers WJ. Cerebral hemodynamic impairment: Methods of measurement and association with stroke risk. Neurology. 1999;53:251–9. 10.1212/wnl.53.2.251 10430410

[25] de Nie AJ, Blankensteijn JD, Visser GH, van der Grond J3, Eikelboom BC. Cerebral blood flow in relation to contralateral carotid disease, an MRA and TCD study. Eur J Vasc Endovasc Surg. 2001;21:220–6. 10.1053/ejvs.2000.1308 11352680

[26] Klijn CJM, Kappelle LJ. Haemodynamic stroke: clinical features, prognosis, and management. Lancet Neurol. 2010;9:1008–17. 10.1016/S1474-4422(10)70185-X 20864053

[27] Caplan LR, Wong KS, Gao S, Hennerici MG. Is hypoperfusion an important cause of strokes? If so, how? Cerebrovasc Dis. 2006;21:145–53. 10.1159/000090791 16401883

[28] Fang H, Song B, Cheng B, Wong KS, Xu YM, Ho SSY, et al Compensatory patterns of collateral flow in stroke patients with unilateral and bilateral carotid stenosis. BMC Neurol. 2016;16:4–9. 10.1186/s12883-015-0516-9 26987874 PMC4797199

[29] Berg P, Saalfeld S, Voß S, Beuing O, Janiga G. A review on the reliability of hemodynamic modeling in intracranial aneurysms: Why computational fluid dynamics alone cannot solve the equation. Neurosurg Focus. 2019;47:1–9. 10.3171/2019.4.FOCUS19181 31261119

[30] Chnafa C, Brina O, Pereira VM, Steinman DA. Better than nothing: A rational approach for minimizing the impact of outflow strategy on cerebrovascular simulations. Am J Neuroradiol. 2018;39:337–43. 10.3174/ajnr.A5484 29269407 PMC7410584

[31] Reymond P, Perren F, Lazeyras F, Stergiopulos N. Patient-specific mean pressure drop in the systemic arterial tree, a comparison between 1-D and 3-D models. J Biomech. 2012;45:2499–505. 10.1016/j.jbiomech.2012.07.020 22884968

[32] Hartkamp MJ, van ver Grond J, van Everdingen KJ, Hillen B, Mali WPTM. Circle of Willis collateral flow investigated by magnetic resonance angiography. Stroke. 1999;30:2671–8. 10.1161/01.str.30.12.2671 10582995

[33] Blankensteijn JD, van der Grond J, Mali WP, Eikelboom BC. Flow volume changes in the major cerebral arteries before and after carotid endarterectomy: An MR angiography study. Eur J Vasc Endovasc Surg. 1997;14:446–50. 10.1016/s1078-5884(97)80122-0 9467518

[34] Loudon C, Tordesillas A. The use of the dimensionless Womersley number to characterize the unsteady nature of internal flow. J Theor Biol. 1998;191:63–78. 10.1006/jtbi.1997.0564 9593657

[35] İdil Soylu A, Ozturk M, Akan H. Can vessel diameters, diameter ratios, and vessel angles predict the development of anterior communicating artery aneurysms: A morphological analysis. J Clin Neurosci. 2019;68:250–5. 10.1016/j.jocn.2019.07.024 31358430

[36] Zurada A, St Gielecki J, Tubbs RS, Loukas M, Zurada-Zielińska A, Maksymowicz W, et al Three-dimensional morphometry of the A1 segment of the anterior cerebral artery with neurosurgical relevance. Neurosurgery. 2010;67:1768–82. 10.1227/NEU.0b013e3181fa7fcb 27759661

[37] Drummond JC. Blood pressure and the brain: How low can you go? Anesth Analg. 2019;128:759–71. 10.1213/ANE.0000000000004034 30883421

[38] Yamauchi H, Higashi T, Kagawa S, Nishii R, Kudo T, Sugimoto K, et al Is misery perfusion still a predictor of stroke in symptomatic major cerebral artery disease? Brain. 2012;135:2515–26. 10.1093/brain/aws131 22637544

[39] Gupta A, Chazen JL, Hartman M, Delgado D, Anumula N, Shao H, et al Cerebrovascular reserve and stroke risk in patients with carotid stenosis or occlusion. A systematic review and meta-analysis. Stroke. 2012;43:2884–91. 10.1161/STROKEAHA.112.663716 23091119 PMC3500140

[40] Bijker J, Persoon S, Peelen L, Moons K, Kalkman C, Kappelle L, et al Intraoperative hypotension and perioperative ischemic stroke after general surgery. A nested case-control study. Anesthesiology. 2012;116 10.1097/ALN.0b013e3182472320 22277949

[41] Chongruksut W, Vaniyapong T, Rerkasem K. Routine or selective carotid artery shunting for carotid endarterectomy (and different methods of monitoring in selective shunting). Cochrane Database Syst Rev. 2014. 10.1002/14651858.CD000190.pub3 PMC7032624 24956204

[42] Ren Y, Chen GZ, Liu Z, Cai Y, Lu GM, Li ZY. Reproducibility of image-based computational models of intracranial aneurysm: A comparison between 3D rotational angiography, CT angiography and MR angiography. Biomed Eng Online. 2016;15:1–14. 10.1186/s12938-016-0163-4 27150439 PMC4858827

[43] Choi CG, Lee DH, Lee JH, Pyun HW, Kang DW, Kwon SU, et al Detection of intracranial atherosclerotic steno-occlusive disease with 3D time-of-flight magnetic resonance angiography with sensitivity encoding at 3T. Am J Neuroradiol. 2007;28:439–46. 17353309 PMC7977826

[44] Igase K, Igase M, Matsubara I, Sadamoto K. Mismatch between TOF MR angiography and CT angiography of the middle cerebral artery may be a critical sign in cerebrovascular dynamics. Yonsei Med J. 2018;59:80–4. 10.3349/ymj.2018.59.1.80 29214780 PMC5725368

[45] Oelerich M, Lentschig MG, Zunker P, Reimer P, Rummeny EJ, Schuierer G. Intracranial vascular stenosis and occlusion: comparison of 3D time-of-flight and 3D phase-contrast MR angiography. Neuroradiology. 1998;40:567–73. 10.1007/s002340050645 9808312

