# Supplementary figures and images for: Middle cerebral artery pressure laterality in patients with symptomatic ICA stenosis
Source: PLoS One. 2021 Jan 8;16(1):e0245337. doi: 10.1371/journal.pone.0245337 (PMC7793245; doi:10.1371/journal.pone.0245337)

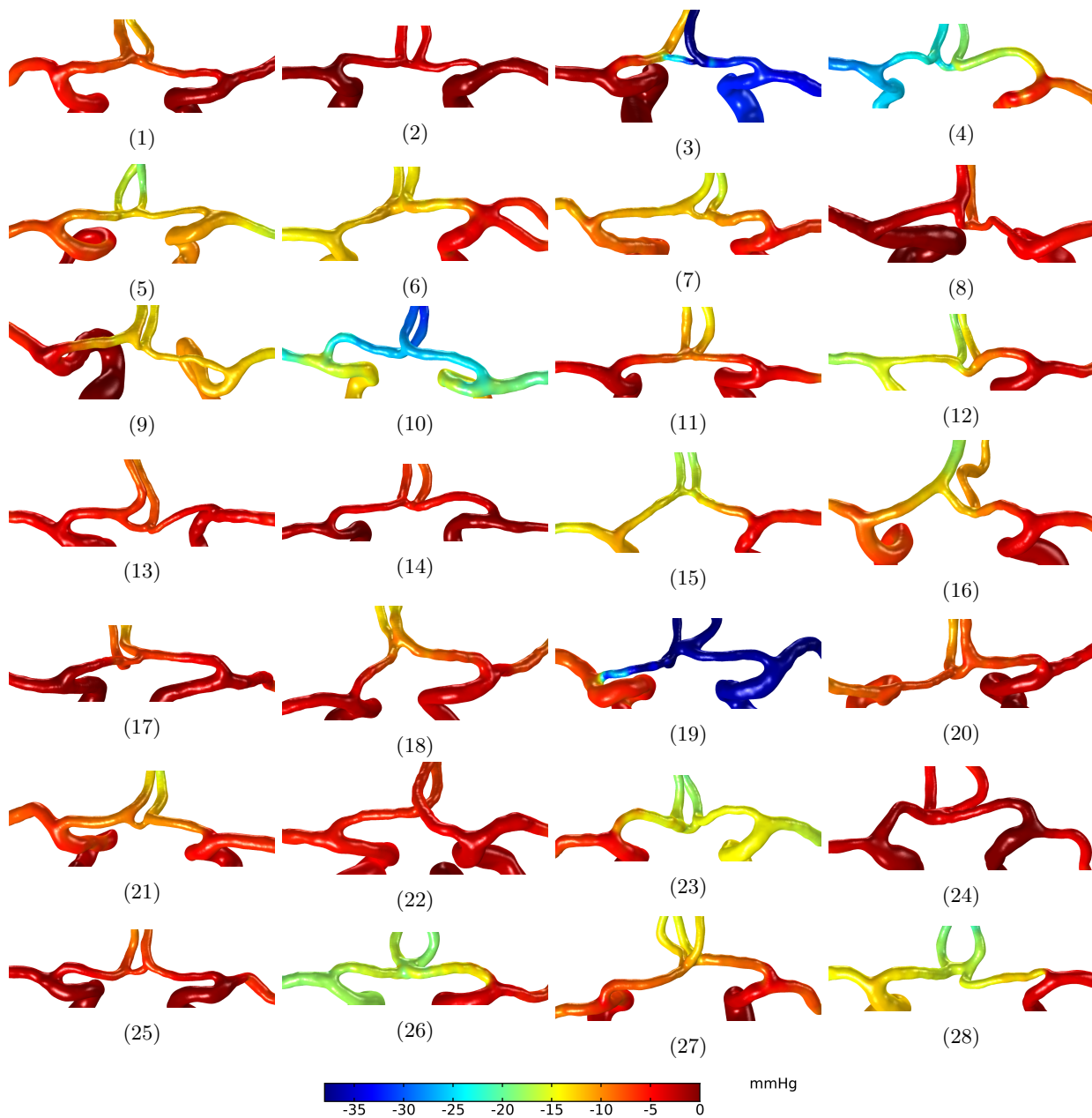

Supplement: S1 Fig — The simulated pressures for each geometry of the 28 patients. The scale might differ between images. (PDF) [file pone.0245337.s002.pdf]
